# Integrating morphological and molecular diversity to develop high-biomass fodder pearl millet composites

**DOI:** 10.3389/fpls.2026.1767075

**Published:** 2026-02-18

**Authors:** Udit Prakash, Vijay Kumar Yadav, Brijesh Kumar Mehta, Krishna Kumar Dwivedi, Dhanapati Keerthana, Rakesh Choudhary, Shweta, Pankaj Kaushal, Shashikumara P.

**Affiliations:** 1 Rani Lakshmi Bai Central Agricultural University, Jhansi, Uttar Pradesh, India; 2 Indian Grassland and Fodder Research Institute, Jhansi, Uttar Pradesh, India; 3 Chandra Shekhar Azad University Agriculture and Technology, Kanpur, Uttar Pradesh, India

**Keywords:** forage pearl millet, genetic diversity, SSR markers, biomass yield, composites

## Abstract

Pearl millet (*Pennisetum glaucum* L. R. Br., syn. *Cenchrus americanus* [L.] Morrone) is a climate-resilient cereal and a vital fodder source in arid and semi-arid regions. Identification and characterization of diverse inbred lines are essential for developing superior forage composites and hybrids with enhanced yield and stress resilience. In this study, 96 fodder pearl millet inbreds along with four checks were evaluated during rainy season 2024 and summer seasons 2025 for 29 morpho-physiological and root architectural traits, complemented by molecular characterization using 46 polymorphic SSR markers. Significant genotypic variation and strong genotype × season interactions were observed for key yield and physiological traits, indicating substantial environmental responsiveness. Correlation analyses identified stem girth, plant height, dry matter yield, and major root traits as major determinants of green fodder yield. Morphological clustering grouped genotypes into five clusters, with maximum divergence between Clusters II and V. SSR analysis detected 203 alleles across 46 loci (average: 5.28 alleles per locus; PIC = 0.62), and population structure analysis resolved six genetic groups highlighting their potential use as heterotic parents. Based on combined phenotypic and molecular diversity, selected inbreds were randomly intermated to develop eight fodder composites. Two composites exhibited 17–20% higher green fodder yield than the best check cultivar. These results demonstrate that integrating morphological and molecular diversity enables effective parental selection and rapid development of superior high-biomass fodder pearl millet composites.

## 1 Introduction

Pearl millet (*Pennisetum glaucum* [L.] R. Br., syn. *Cenchrus americanus* [L.] Morrone) is a short-duration, warm-season annual cereal belonging to the *Poaceae* family and *Panicoideae* subfamily. The protogynous flowering habit of pearl millet encourages natural cross-pollination primarily by wind (Jauhar and Hanna, 1998).

Globally, pearl millet is cultivated over 30 million hectares in more than 30 countries, predominantly in Asia (>10 million ha), Africa (~18 million ha), and South America (>2 million ha) (Deevi et al., 2024). India is the largest producer, accounting for approximately 38.4% of global production at 10.86 million tons from 7.21 million hectares. The major producing states are Rajasthan, Uttar Pradesh, Gujarat, Haryana, and Madhya Pradesh, collectively contributing over 90% of India’s total production (ICAR-AICRP on Pearl millet). Fodder Pearl millet is cultivated approximately about 0.9 Mha in India and primely cultivated during both Rainy and Summer season.

Pearl millet surpasses other common forage crops like maize due to its superior tolerance to heat, drought, and saline or poor soils. Its grain is nutritionally rich, containing protein (11.6 g), carbohydrates (67.5 g), fat (5.0 g), calcium (42 mg), phosphorus (296 mg), iron (10.3 mg), and zinc (3.10 mg) per 100 grams (Hassan et al., 2014). Free from prussic and hydrocyanic acids, pearl millet forage is more digestible and nutritious than sorghum or Sudan grass (Babiker et al., 2024). It yields substantial biomass with nutritive value suitable for silage, hay, green fodder, and stover production. The development of multicut forage pearl millet cultivars has made forage pearl millet a popular fodder crops in India. Multi-cut cultivars can produce upto 800 q/ha green forage yield under rainfed or irrigated conditions, with dry matter content of 15-20%, crude protein of 7-10%, and digestibility of 50-55% (Kaushal et al., 2024). Efforts by pearl millet scientists focus mainly on grain production, yet forage potential remains understudied. In fodder pearl millet, area under cultivation is increasing especially during summer season in India due to its ability to survive at high temperature, and provide long duration green fodder through its multi-cut ability (Ponnaiah et al., 2019).

Morphological evaluation alone is often insufficient for reliable genetic characterization in pearl millet due to strong environmental influence and its predominantly cross-pollinated nature. Many researchers around the globe utilized agro-morphological and/or molecular markers in identification of genetically diverse and superior pearl millet inbreds with the aim to develop heterotic hybrids and composites (Chandra Shekara et al., 2007). The majority of the pearl millet breeding programmes in India has been focused on improving the grain yield, quality and disease resistance (Patil et al., 2021). Most of the cultivated varieties of fodder pearl millet in India are OPVs and hybrid breeding for fodder is lagging behind as compared to hybrid breeding for grain. So, there is enormous scope for breeding hybrid cultivars by exploiting heterosis, based on ago-morphological and molecular diversity among fodder pearl millet inbreds. Molecular markers therefore provide an effective complementary approach for assessing genetic diversity, population structure, and relatedness among inbred lines. In the present study, simple sequence repeat (SSR) markers were employed primarily to quantify genome-wide diversity and genetic divergence, owing to their co-dominant inheritance, reproducibility, and proven utility in diversity analysis of pearl millet germplasm (Govindaraj et al., 2018; Puttamadanayaka et al., 2020). Although trait-specific functional markers such as SNPs, KASP assays, or haplotype-based markers are more suitable for marker-assisted selection of defined loci (Shashikumara et al., 2026), such markers are not yet available for many complex biomass- and root-related traits in fodder pearl millet. Therefore, SSR markers were used here as neutral markers to support diversity studies and parental selection, rather than for direct marker-assisted selection. Integration of molecular diversity with detailed phenotypic evaluation for biomass component traits, root traits and physiological traits was used to guide the identification of genetically divergent and agronomically superior inbreds for composite development.

The Open pollinated variety (OPVs) are forage pearl millet cultivars currently cultivated in India which are mostly developed through selection from germplasm lines. We need exploit heterosis in terms of developing composites and hybrids. With this background we have developed many fodder pearl millet inbreds. Here, our aim is to break the yield barriers in fodder pearl millet by developing composites, which are superior yielding over existing OPVs by exploiting these inbreds. Further, the diverse inbreds could be utilized in future for developing fodder pearl millet hybrids. In this study, we have evaluated the newly developed inbreds for morpho-physiological and root architectural traits for two seasons and did molecular diversity analysis to identify potential inbreds, which are diverse apart to develop high yielding fodder pearl millet composites. The developed composites were evaluated for green forage yield and in future will be submitted for varietal trial to AICRP (Forage Crops & Utilization) for multi-location testing across different agro-climatic zones of India.

## 2 Materials and methods

### 2.1 Plant material

The experimental material comprised of 96 forage pearl millet inbreds and four commercial checks (Nutrifeed, Rajka Bajri, Giant Bajra, and BAIF Bajra-1) laid out in an alpha lattice design with two replications (**Supplementary Table S1**). Genotypes were planted in 10 blocks, each consisting of 2-meter-long rows with a spacing of 30 cm between rows and 10 cm between plants. The present study on forage pearl millet (*Pennisetum glaucum* L. R. Br.) was conducted during the rainy and summer seasons at the ICAR–Indian Grassland and Fodder Research Institute (ICAR–IGFRI), Jhansi, Uttar Pradesh, India. The experimental site is situated on a plateau at an elevation of 285 m above sea level (25°51′N, 78°53'E), characterized by an average annual rainfall of 870 mm and a mean temperature of 25.8°C, with pronounced seasonal extremes due to its topography. All the recommended package of practices and plant protection measures were applied to obtain optimum crop stand.

### 2.2 Morphological and physiological data collection

Morphological and physiological traits were recorded from five randomly selected plants per genotype. Morphological traits were recorded for plant height, leaf length and width, leaf and flag leaf area (Epson Perfection V700 scanner analyzed *via* BioVis PSM software), spike length and girth (measured at the middle of the spike on the main tiller using digital vernier caliper), Total Soluble Solids (Zeiss handheld Refractometer), days to 50% flowering, productive tillers per plant, stem girth (measured at third internode from base using digital vernier calliper), regeneration percentage (biomass after regrowth, i.e., green fodder yield at second cut and third cut, divided by, biomass before cutting, i.e., green fodder yield at first cut). The first cut is done at 50 days after sowing (DOS), second cut at 80 DAS and third cut 110 DOS, leaf-to-stem ratio, days to maturity, node count per plant, green fodder yield, and dry fodder yield (DFY) (dry fodder yield 1: DFY after first cut, dry fodder yield 2: DFY after second cut and dry fodder yield 2: DFY after third cut). Regeneration percentage was calculated as:


$$\text{Regeneration }\%=\frac{\;Biomass\;after\;regrowth}{Biomass\;before\;cutting}\times 100$$

Physiological traits such as total chlorophyll content (measured using apogee chlorophyll meter for three leaves per plant) and canopy temperature (infrared thermometer) were recorded. Root architectural traits such as total root length, root tips, forks, segments, average root diameter, maximum and minimum root diameter, root volume, and primary root length were quantified using scanned images (200 dpi resolution) using an Epson Perfection V700 scanner analyzed *via* BioVis PSM software.

### 2.3 Experimental design and statistical analysis

Pooled analysis of variance (ANOVA) was performed for data recorded across multiple cuts and two seasons to test the significance of differences among genotypes. The field experiment was conducted using an alpha lattice design, and REML ANOVA and Violin plot analysis were carried out in R software (version 4.4.1 https://www.R-project.org/) using the “agricolae” package (https://CRAN.R-project.org/package=agricolae), employing the PBIB.test function suitable for alpha lattice analysis. Genetic variability parameters, including genotypic variance, phenotypic variance, genotypic and phenotypic coefficients of variation, broad-sense heritability (H²), genetic advance (GA), and genetic advance as a percentage of the mean (GAM), were estimated using the TBA (Tool for Biometrical Analysis) package (https://CRAN.R-project.org/package=TBA). The “metan” package in R software version 4.4.1 (Olivoto and Lúcio, 2020) was used to calculate the Pearson’s correlation coefficients between the morphological, physiological, and root architectural traits. Cluster analysis was carried out using the “factoextra, tidyverse, cluster, and ggplot2” packages in R software version 4.4.1 (Wickham et al., 2019). Principal component analysis was performed using the “factoMiner and factoextra” packages in R software version 4.4.1 (Lê et al., 2008; Kassambara and Mundt, 2021).

### 2.4 Molecular characterization

Genomic DNA was extracted from young leaf tissue using a modified CTAB protocol (Doyle and Doyle, 1990). The extracted DNA was treated with RNase, purified, and quantified using a Nanodrop spectrophotometer. DNA integrity was confirmed by electrophoresis on 0.8% agarose gels stained with ethidium bromide and visualized under ultraviolet light. A total of 46 SSR primers (**Supplementary Table S2**) were used for molecular characterization. Primers were diluted to 10 μM working concentrations, and PCR amplification was performed in 10 μL reaction volumes containing 13 ng of template DNA, 10 pmol of each primer, 3.5 μL of master mix, and nuclease-free water. The PCR protocol included an initial denaturation at 94°C for 4 min, followed by 30 cycles of denaturation at 94°C for 30 s, annealing at 55–58.5°C for 30 s, and extension at 72°C for 1 min, with a final extension at 72°C for 10 min. Amplified products were resolved on 3% agarose gels using 1× TAE buffer, with a 100 bp DNA ladder as reference for fragment size estimation. Banding patterns were scored as present (1) or absent (0) for binary data analysis.

Population structure was inferred using population structure analysis software STRUCTURE v2.3.4 (Pritchard et al., 2000). Genetic diversity parameters were estimated using PowerMarker v3.25, and analysis of molecular variance (AMOVA) was conducted using GenAlex v6.4 (Peakall and Smouse, 2006) Dendrogram was constructed using DARwin software (Peakall and Smouse, 2006) based on the unweighted neighbor-joining method.

### 2.5 Development and evaluation of composites

For composite development, total 100 seeds of each 5 to 6 selected inbreds identified through molecular and morphological diversity analyses were mixed in equal proportions and sown together under isolation during summer season 2025, following standard isolation distances and recommended agronomic practices to ensure random mating (**Supplementary Table S4**). After harvesting, eight composite seed lots were generated. These composites, along with three check cultivars, were evaluated during Rainy season 2026 in a Randomized Block Design (RBD) with three replications. Each plot measured 3 × 6 meters, with a row spacing of 30 cm. Data were recorded for key traits, including green forage yield, dry matter yield, plant height and days to 50% flowering.

## 3 Results

### 3.1 Phenotypic variation

Analysis of variance (ANOVA) revealed highly significant differences among the 96 forage pearl millet inbreds and four check cultivars for agro-morphological, physiological, and root architectural traits, indicating substantial genetic variability in the experimental material (**Table 1**). During both the rainy (Rainy 2024) and summer (Summer 2025) seasons, genotypic effects were highly significant (p < 0.01) for most traits. Further, the REML-based ANOVA also indicated highly significant genotypic effects for most of the such as plant height, leaf traits, spike traits, fodder yield, chlorophyll content, and detailed root parameters showed strong genetic control relative to residual variation (**Supplementary Table S3**). In contrast, days to maturity and leaf-to-stem ratio were non-significant, suggesting limited genotypic differences for these traits under the tested conditions. Overall, the results confirm ample scope for effective selection and genetic improvement, particularly for yield, stress-adaptive, and root-related traits.

**Table 1:** ANOVA for the 29 studied traits in pearl millet inbreds.

	Parameters	Source	Replication	Treatment (adj.)	Block (within rep.)	Error
		df	1	99	9	90
Mean Sum of Square	Plant height	*Rainy*2024	413.91	94.69**	37.05	19.67
		*Summer* 2025	2732.75**	1417.65**	414.58	250.19
	Leaf length	*Rainy*2024	60.79	2097.39**	874.27	609.52
		*Summer* 2025	1995.41**	132.60**	49.55	37.09
	Leaf width	*Rainy*2024	0.01	1.33**	0.16	0.09
		*Summer* 2025	0.24	0.64**	0.06	0.07
	Flag leaf area	*Rainy*2024	0.68	2987.13**	48.79	49.83
		*Summer* 2025	113.97	2970.49**	50.52	70.93
	Leaf area	*Rainy*2024	128.06	3578.04**	351.61	283.58
		*Summer* 2025	479.60	3407.50**	423.40*	196.00
	Spike Length	*Rainy*2024	0.05	45.32**	4.78	7.38
		*Summer* 2025	3.25	36.06**	2.79	4.23
	Spike Girth	*Rainy*2024	14.64	39.70**	3.32	6.06
		*Summer* 2025	102.28**	34.25**	2.64	8.76
	Days to 50% flowering	*Rainy*2024	0.18	26.26**	0.36	0.45
		*Summer* 2025	147.92**	20.00**	10.95**	3.45
	Days to Maturity	*Rainy*2024	0.98	2.73**	2.01**	0.64
		*Summer* 2025	9.24**	3.85**	1.22	0.88
	Productive tillers per plant	*Rainy*2024	0.52	10.06**	0.66	1.00
		*Summer* 2025	4.76**	10.09**	0.58	0.61
	Stem Girth	*Rainy*2024	0.63	7.98**	0.77	1.48
		*Summer* 2025	0.27	10.31**	0.06	0.12
	Leaf to Stem Ratio	*Rainy*2024	0.00	0.02**	0.00	0.00
		*Summer* 2025	0.01	0.09**	0.01	0.01
	Nodes/plant	*Rainy*2024	0.92	6.82**	0.44	0.78
		*Summer* 2025	7.81**	1.76**	0.67	0.83
	Green Fodder Yield 1	*Rainy*2024	0.19	12.98**	0.19	0.30
		*Summer* 2025	2.77**	8.60**	0.29*	0.14
	Green Fodder Yield 2	*Rainy*2024	0.42*	4.59**	0.04	0.08
		*Summer* 2025	0.00	3.05**	0.01	0.05
	Green Fodder Yield 3	*Rainy*2024	0.03	1.24**	0.01	0.02
		*Summer* 2025	0.05	1.01**	0.02	0.03
	Dry Fodder Yield 1	*Rainy*2024	0.04*	0.85**	0.02	0.01
		*Summer* 2025	0.01	0.43**	0.00	0.00
	Dry Fodder Yield 2	*Rainy*2024	0.02**	0.15**	0.00	0.00
		*Summer* 2025	0.00*	0.09**	0.00	0.00
	Dry Fodder Yield 3	*Rainy*2024	0.00*	0.06**	0.00	0.00
		*Summer* 2025	0.00	0.04**	0.00	0.00
	Regeneration 1%	*Rainy*2024	540.48*	2071.25**	75.69	113.98
		*Summer* 2025	847.94*	2261.87**	39.95	144.24
	Regeneration 2%	*Rainy*2024	150.25	345.72**	115.53*	54.53
		*Summer* 2025	748.30*	518.78**	46.59	114.98
	Total Chlorophyll Content	*Rainy*2024	0.15	146.08**	19.98	15.87
		*Summer* 2025	3.28	131.44**	4.49	16.46
	Canopy Temperature	*Rainy*2024	33.55	16.15	18.79	13.76
		*Summer* 2025	0.25	1.01**	0.18	0.15
	Total Soluble Solids	*Rainy*2024	0.00	2.27**	0.18	0.13
		*Summer* 2025	0.01	2.48**	0.01	0.01
	Root Projected Area	*Rainy*2024	0.53	416.36**	7.66	9.80
		*Summer* 2025	17.23	401.86**	5.69	9.51
	Total Root Length	*Rainy*2024	5876.80	11849.87**	144.72**	252.82
		*Summer* 2025	19.80	12181.40**	342.30	380.30
	Root Tips	*Rainy*2024	35390.50	34907.57	1433.01	989.24
		*Summer* 2025	4743*	34257.00**	896.00	1104.00
	Forks	*Rainy*2024	58479.60	25298.63**	1166.24**	993.39
		*Summer* 2025	1501.50*	3029.30**	34.10	25.00
	Segments	*Rainy*2024	399311.42	149213.04**	958.48**	1976.22
		*Summer* 2025	1205.00	168559.00**	10442.00	7944.00
	Minimum Root Diameter	*Rainy*2024	0.00	0.00	0.00	0.00
		*Summer* 2025	0.00	0.00*	0.00	0.00
	Maximum Root Diameter	*Rainy*2024	0.19*	0.52**	0.02	0.03
		*Summer* 2025	0.14*	0.61**	0.02	0.02
	Average Root Diameter	*Rainy*2024	0.03**	0.02**	0.01**	0.00
		*Summer* 2025	0.00	0.02**	0.00	0.00
	Root Volume	*Rainy*2024	80.95**	0.67	0.08	0.27
		*Summer* 2025	0.67	1106.00**	51.70**	53.08**
	Primary Root Length	*Rainy*2024	0.08	51.7**	0.09	0.18
		*Summer* 2025	0.27	53.08**	1.30	4.12

*Significant at 5% level; **Significant at 1% level.

Across seasons, the pattern of genotypic significance remained largely consistent, demonstrating stable expression of genetic variability under both rainy and summer environments. The similarity in magnitude and significance of genotype mean squares between seasons indicates that seasonal influences did not markedly alter relative genotypic performance. Minor season-specific variations observed for a few traits were attributed to environmental fluctuations rather than true changes in genotype ranking. Overall, the ANOVA results presented in **Table 1** and **Supplementary Table S7** confirm broad genetic diversity coupled with stable genotypic performance across seasons, supporting the robustness of multi-trait selection for forage pearl millet improvement.

### 3.2 Morphological trait characterization: mean performance and genetic variability

The overall mean performance of 96 forage pearl millet inbreds across rainy and summer seasons revealed substantial variability in morphological, physiological, and root traits (**Table 2**). Average plant height was 184.15 cm, leaf length 65.54 cm, and leaf width 3.54 cm. Flag leaf area averaged 74.78 cm², while total leaf area was 171.91 cm². Spike length and girth recorded mean values of 25.07 cm and 23.76 cm, respectively. Days to 50% flowering and maturity averaged 62.44 and 125.42 days, respectively. Genotypes produced an average of 8.74 productive tillers, with stem girth at 12.78 mm, leaf-to-stem ratio at 0.56, and node count at 8.61. Mean green fodder yield was 6.67 kg/plot and dry fodder yield 1.22 kg/plot. Physiological traits showed average chlorophyll content of 36.44 µmol/m², regeneration percentages of 62.75% (cut 1) and 58.94% (cut 2), canopy temperature of 29.97°C, and total soluble solids of 3.62°Brix. Root traits revealed an average projected area of 25.30 cm², total root length of 186.21 cm, 321.41 root tips, 385.71 forks, 633.70 segments, maximum root diameter of 1.46 cm, average diameter of 0.24 cm, root volume of 21.64 cm³, and primary root length of 22.56 cm.

**Table 2:** Genetic variability parameters in Pearl millet Inbreds.

Parameter	Season	Grand mean	Phenotypic variance	Genotypic variance	P.C.V.(%)	G.C.V.(%)	Heritability (bs) ( % )	Genetic Advance	GAM	S.E. (mean)	Range
Plant Height	*S1*	172.15	1365.49	731.91	21.47	15.72	53.60	40.80	23.70	17.80	113.17-269.00
	*S2*	196.15	841.39	576.26	14.79	12.24	68.49	40.93	20.86	11.51	131.00-253.67
Leaf Length	*S1*	62.48	105.76	57.51	16.46	12.14	54.38	11.52	18.44	4.91	39.50-86.33
	*S2*	68.62	85.41	47.19	13.47	10.01	55.25	10.52	15.33	4.37	44.84-87.67
Leaf Width	*S1*	3.78	0.71	0.62	22.29	20.78	86.87	1.51	39.89	0.22	1.67-5.84
	*S2*	3.30	0.36	0.29	18.08	16.20	80.29	0.99	29.90	0.19	2.17-4.67
Flag Leaf Area	*S1*	76.94	1518.43	1468.70	50.65	49.81	96.73	77.64	100.92	4.99	23.84-206.34
	*S2*	72.63	1519.79	1450.71	53.68	52.45	95.46	76.66	105.55	5.88	23.50-187.84
Leaf Area	*S1*	172.10	1933.91	1644.14	25.55	23.56	85.02	77.02	44.75	12.04	100.83-299.00
	*S2*	171.71	1812.06	1595.39	24.79	23.26	88.04	77.21	44.96	10.41	105.00-278.84
Spike Length	*S1*	25.63	26.24	19.09	19.98	17.05	72.77	7.68	29.96	1.89	13.84-39.00
	*S2*	24.51	22.42	13.22	19.32	14.84	58.98	5.75	23.48	2.14	13.67-33.00
Spike Girth	*S1*	24.25	22.76	16.95	19.67	16.97	74.47	7.32	30.17	1.71	14.50-36.67
	*S2*	23.27	21.23	13.03	19.80	15.51	61.37	5.82	25.03	2.03	13.83-32.34
Days to 50% Flowering	*S1*	62.53	13.35	12.91	5.84	5.75	96.69	7.28	11.64	0.47	51.50-67.50
	*S2*	62.35	12.07	7.94	5.57	4.52	65.76	4.71	7.55	1.44	54.00-66.50
Days to Maturity	*S1*	121.04	1.75	0.98	1.09	0.82	56.15	1.53	1.27	0.62	118.50-125.50
	*S2*	129.80	2.38	1.47	1.19	0.93	61.70	1.96	1.51	0.68	127.00-134.50
Productive Tillers/p	*S1*	8.83	5.51	4.55	26.58	24.14	82.50	3.99	45.17	0.70	5.17-18.17
	*S2*	8.65	5.35	4.74	26.76	25.19	88.62	4.22	48.85	0.55	5.33-16.50
Stem Girth	*S1*	12.26	4.70	3.28	17.68	14.78	69.83	3.12	25.44	0.84	8.34-17.00
	*S2*	13.29	5.22	5.10	17.18	17.00	97.86	4.60	34.64	0.24	6.84-18.67
Leaf to Stem Ratio	*S1*	0.40	0.01	0.01	29.95	27.98	87.23	0.21	53.83	0.03	0.16-0.80
	*S2*	0.72	0.05	0.05	31.13	29.37	89.00	0.41	57.09	0.05	0.32-1.50
Nodes per plant	*S1*	9.62	3.79	3.04	20.23	18.11	80.17	3.21	33.41	0.61	6.34-16.34
	*S2*	7.59	1.29	0.48	14.97	9.09	36.87	0.86	11.37	0.64	5.00-9.52
Green Fodder Yield	*S1*	7.31	19.23	18.81	59.99	59.33	97.80	8.83	120.88	0.46	1.59-36.63
	*S2*	6.03	13.18	12.91	60.20	59.58	97.95	7.32	121.46	0.37	1.35-31.01
Dry Fodder Yield	*S1*	1.35	1.01	0.99	74.68	74.01	98.23	2.03	151.11	0.09	0.29-9.16
	*S2*	1.08	0.55	0.54	68.92	68.67	99.26	1.52	140.93	0.04	0.23-6.77
Total Chlorophyll Content	*S1*	36.76	81.16	64.92	24.51	21.92	79.99	14.85	40.38	2.85	19.67-60.50
	*S2*	36.12	73.41	58.04	23.72	21.10	79.06	13.95	38.64	2.77	20.17-57.67
Regeneration% 1	*S1*	63.48	1090.86	980.36	52.03	49.32	89.87	61.15	96.32	7.43	13.55-144.27
	*S2*	62.82	1198.31	1063.55	55.11	51.92	88.75	63.29	100.76	8.21	11.35-194.70
Regeneration% 2	*S1*	52.88	202.89	142.82	26.94	22.60	70.39	20.66	39.06	5.48	9.53-79.56
	*S2*	65.14	313.77	205.01	27.19	21.98	65.34	23.84	36.60	7.37	18.50-113.35
Canopy Temperature	*S1*	29.47	15.19	0.97	13.22	3.34	6.36	0.51	1.73	2.67	21.87-35.88
	*S2*	30.46	0.58	0.43	2.51	2.16	74.07	1.16	3.82	0.28	29.30-32.65
Total Soluble Solids	*S1*	3.66	1.20	1.07	29.99	28.25	88.73	2.01	54.82	0.26	2.00-10.75
	*S2*	3.58	1.25	1.24	31.17	31.06	99.32	2.28	63.77	0.07	2.00-10.75
Root Projected Area	*S1*	25.60	212.99	203.38	57.02	55.71	95.49	28.71	112.15	2.19	2.67-68.59
	*S2*	24.99	205.51	196.35	57.36	56.07	95.54	28.22	112.89	2.14	2.78-69.82
Total Root Length	*S1*	183.29	6046.43	5803.44	42.42	41.56	95.98	153.75	83.88	11.02	26.32-481.11
	*S2*	189.13	6279.09	5902.28	41.90	40.62	94.00	153.44	81.13	13.73	28.81-490.98
Root Tips	*S1*	318.32	17968.58	16939.00	42.11	40.89	94.27	260.31	81.78	22.69	44.97-857.93
	*S2*	324.49	17671.42	16586.07	40.97	39.69	93.86	257.03	79.21	23.30	53.00-809.50
Root Forks	*S1*	376.60	13153.87	12144.77	30.45	29.26	92.33	218.14	57.92	22.46	67.05-529.63
	*S2*	394.82	15132.56	15106.71	31.16	31.13	99.83	252.98	64.07	3.60	63.00-556.00
Segments	*S1*	611.93	75548.37	73664.67	44.92	44.35	97.51	552.10	90.22	30.69	103.92-1726.88
	*S2*	655.46	88365.11	80193.97	45.35	43.20	90.75	555.74	84.79	63.92	96.50-1742.00
Maximum Root Diameter	*S1*	1.45	0.27	0.25	36.13	34.24	89.83	0.97	66.85	0.12	0.45-2.66
	*S2*	1.47	0.32	0.30	38.30	36.85	92.62	1.08	73.06	0.11	0.45-2.87
Average Root Diameter	*S1*	0.23	0.01	0.01	43.85	42.77	95.10	0.20	85.93	0.02	0.00-0.45
	*S2*	0.25	0.01	0.01	45.18	45.18	100.00	0.23	93.05	0.01	0.00-0.49
Estimated Root Volume	*S1*	21.07	479.17	473.94	103.90	103.33	98.91	44.60	211.70	1.62	0.63-81.69
	*S2*	22.20	553.18	552.82	105.95	105.92	99.94	48.42	218.12	0.42	0.57-89.80
Primary Root Length	*S1*	22.53	25.94	25.77	22.61	22.53	99.34	10.42	46.27	0.29	11.96-37.20
	*S2*	22.59	28.48	24.61	23.63	21.97	86.42	9.50	42.06	1.39	12.90-37.41

S1-Rainy season, 2024; S2-Summer season, 2025.

**Figure 1:**
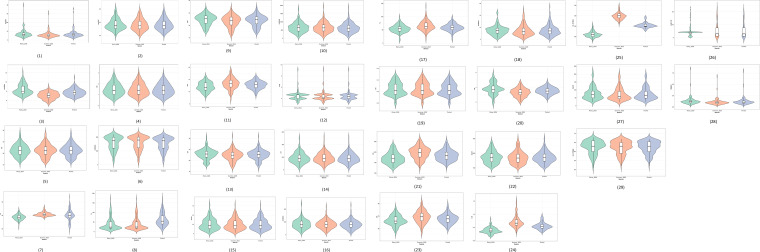
Violin plot depicting the morpho-physiological trait variation of Peal millet inbreds.

The magnitude of genetic variability differed among trait groups, reflecting their relative contribution to fodder productivity (**Table 2**). Biomass-related traits such as green fodder yield (GFY) and dry fodder yield (DFY), along with several root architectural traits, exhibited high genotypic and phenotypic coefficients of variation coupled with high heritability. In contrast, phenological traits such as days to flowering and maturity showed lower variability, indicating tighter genetic control. Root traits consistently displayed high heritability and substantial genetic advance, suggesting strong additive genetic effects and their suitability for indirect selection. The combination of high heritability and moderate-to-high genetic advance for key yield-associated traits highlights the effectiveness of selection for improving forage biomass in pearl millet. **Figure 1** presents the violin plot analysis indicates high phenotypic variation among genotypes for complex traits such as root architectural parameters (total root length, root tips, forks, segments), green fodder yield, dry fodder yield, and leaf area, as evidenced by wider distributions and longer tails. These traits show substantial dispersion, reflecting broad genetic diversity and greater scope for selection. In contrast, low variation was observed for days to maturity, leaf-to-stem ratio and canopy temperature which displayed narrower distributions and compact interquartile ranges. Moderate variability was evident for plant height, spike traits, and chlorophyll content. Overall, the results highlight that yield- and root-related traits contribute most to genotypic differentiation, whereas phenological stability is relatively high across genotypes.

### 3.3 Trait associations governing fodder yield among inbreds

**Figure 2:**
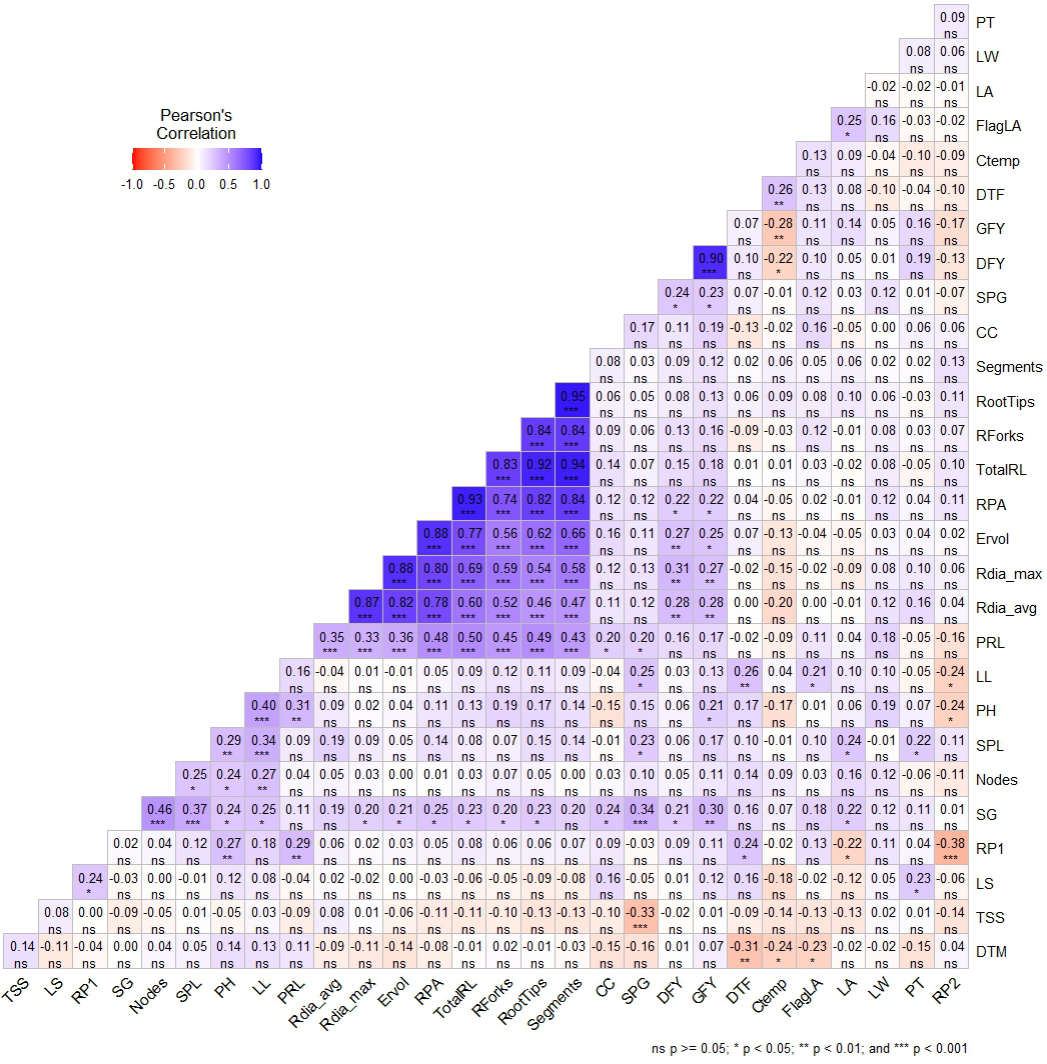
Pearson’s correlation heatmap showing correlation between 29 traits among pearl millet inbreds.

Correlation analysis revealed significant associations among key morphological and root traits contributing to green fodder yield (GFY) and dry fodder yield (DFY) across seasons (**Figure 2**). GFY exhibited positive correlations with stem girth (r = 0.30), PH (r = 0.20), root projection area (r = 0.25), maximum root diameter (r = 0.27), average root diameter (r = 0.28), estimated root volume (r = 0.25), and stem parenchyma girth (SPG; r = 0.23). A strong positive association was recorded between GFY and DFY (r = 0.90), indicating that genotypes with higher green biomass consistently produced greater dry matter. Notably, GFY showed a significant negative correlation with canopy temperature (r = –0.28), suggesting that cooler canopies were associated with superior fodder productivity. DFY demonstrated a correlation pattern similar to GFY, showing positive associations with stem girth (r = 0.21), root projection area (r = 0.22), maximum root diameter (r = 0.31), average root diameter (r = 0.28), estimated root volume (r = 0.27), and SPG (r = 0.24). A significant negative relationship with canopy temperature (r = –0.22) further confirmed the importance of cooler canopy conditions for enhanced biomass accumulation. Overall, the results highlight that both shoot vigor and root system robustness, coupled with lower canopy temperature, play critical roles in determining fodder yield in pearl millet.

### 3.4 Multivariate analysis identifies key traits and superior genotypes

**Figure 3:**
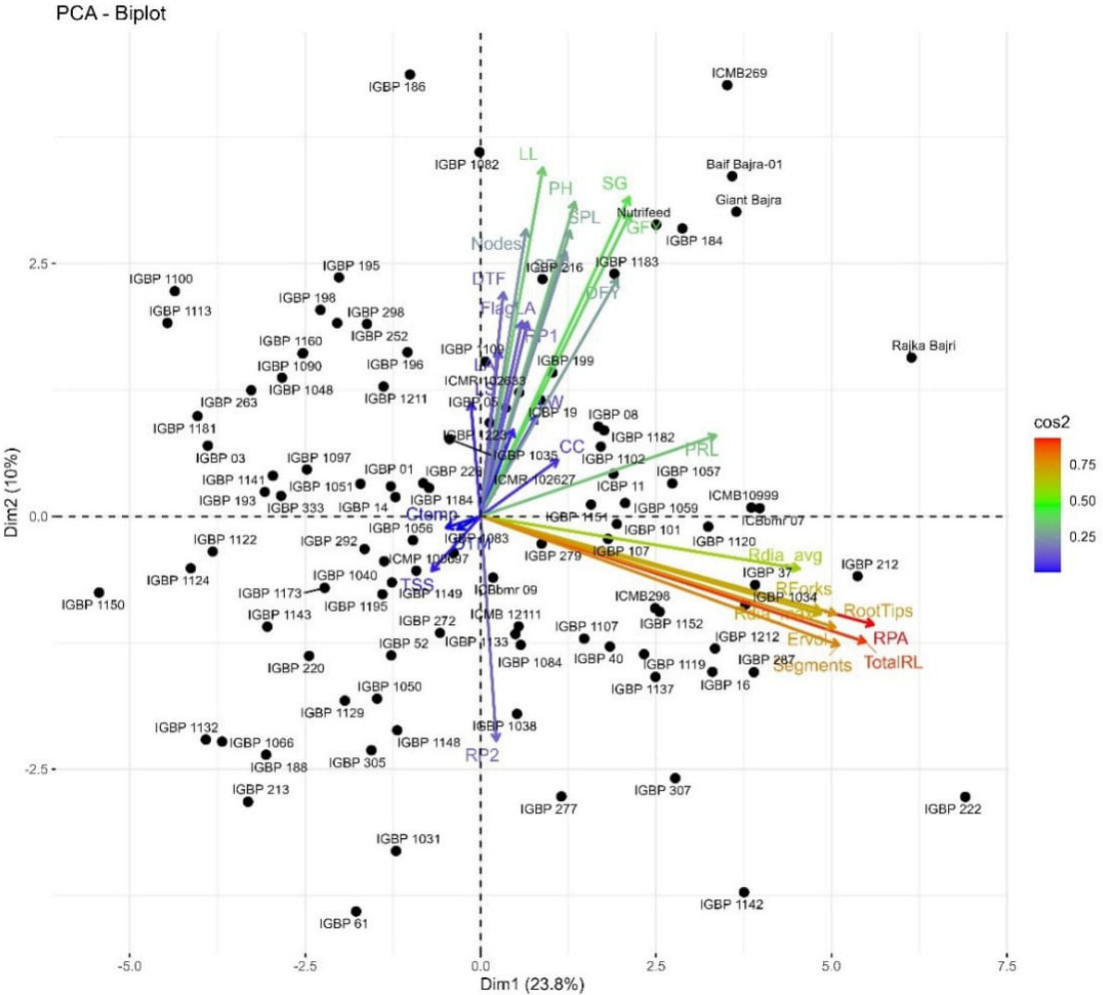
Principal component analysis (PCA) biplot depicting multivariate trait relationships and genotypic diversity among evaluated pearl millet inbreds.

**Figure 4:**
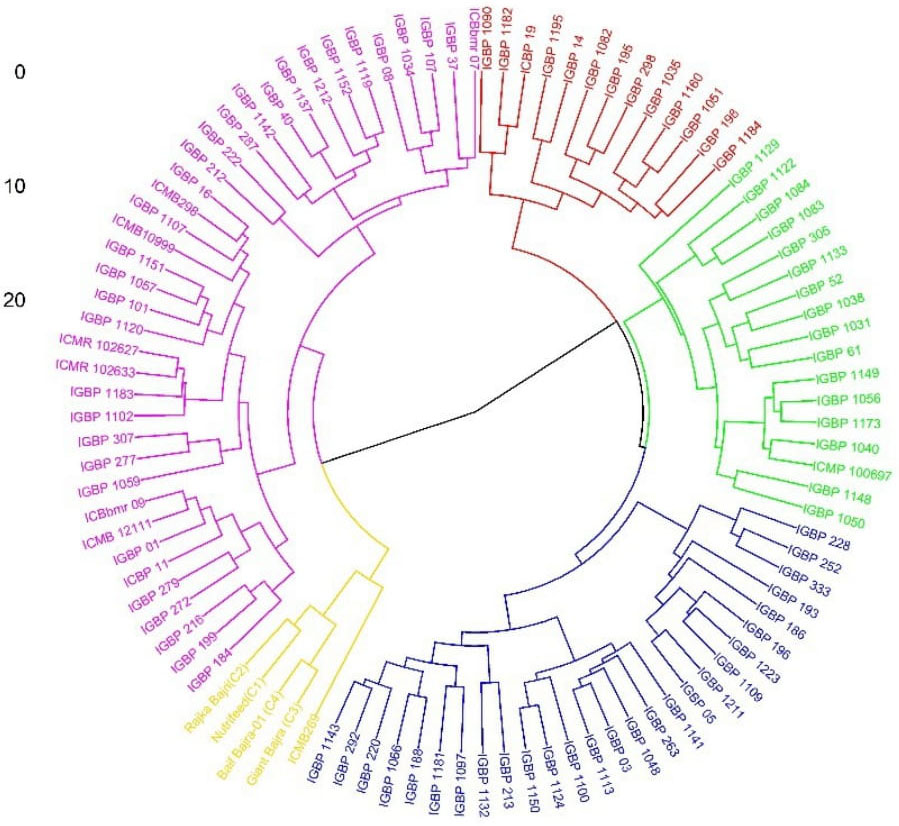
Dendrogram based on Euclidean distance in the studied genotypes.

The PCA biplot integrating traits and genotypes clearly discriminated genotypes based on multivariate trait associations (**Figure 3**). PC1 was largely driven by root architectural traits, whereas PC2 reflected shoot, physiological, and yield-related traits, enabling functional separation of genotypes. Genotypes such as Raika Bajri, Baif Bajra-01, Giant Bajra, IGBP212, IGBP122, IGBP1043, IGBP287, and IGBP16 were positioned in the quadrant jointly influenced by root and yield trait vectors, indicating a favorable combination of below- and above-ground traits. In contrast, several IGBP lines clustered near the origin, reflecting average and stable performance with limited contribution to overall variability. Correlation analysis supported strong positive associations among root traits and between root traits and fodder yield, reinforcing their importance in selection. Overall, the PCA–correlation framework enabled effective identification of superior and contrasting genotypes for use in breeding programs targeting productivity and stress adaptation. Cluster analysis grouped genotypes into five distinct clusters, aligning with PCA patterns and indicating clear multivariate differentiation useful for selecting parents for biomass and stress-resilient breeding. The genotypes were grouped into five distinct clusters (**Table 3**; **Figure 4**). Large inter-cluster distances, particularly between Clusters II and V, indicate substantial divergence and provide opportunities for combining complementary trait profiles. These results demonstrate that multivariate analyses effectively identify both key traits and contrasting genotype groups relevant for breeding.

**Table 3:** Classification of pearl millet Inbreds on the basis of Euclidean distance.

S. No	Cluster number	Cluster colour	Inbreds
1	Cluster I	Pink	IGBP 184, IGBP 199, IGBP 216, IGBP 272, IGBP 279, ICBP 11, IGBP 01, ICMB 12111, ICBbmr 09, IGBP 1059, IGBP 277, IGBP 307, IGBP 1102, IGBP 1183, ICMR 102633, ICMR 102627, IGBP 1120, IGBP 101, IGBP 1057, IGBP 1151, ICMB10999, IGBP 1107, ICMB298, IGBP 16, IGBP 212, IGBP 222, IGBP 287, IGBP 1142, IGBP 40, IGBP 1137, IGBP 1212, IGBP 1152, IGBP 1119, IGBP 08, IGBP 1034, IGBP 107, IGBP 37, ICBbmr 07
2	Cluster II	Blue	IGBP 228, IGBP 252, IGBP 333, IGBP 193, IGBP 186, IGBP 196, IGBP 1223, IGBP 1109, IGBP 1211, IGBP 05, IGBP 1141, IGBP 263, IGBP 1048, IGBP 03, IGBP 1113, IGBP 1100, IGBP 1124, IGBP 1150, IGBP 213, IGBP 1132, IGBP 1097, IGBP 1181, IGBP 188, IGBP 1066, IGBP 220, IGBP 292, IGBP 1143
3	Cluster III	Red	IGBP 1090, IGBP 1182, ICBP 19, IGBP 1195, IGBP 14, IGBP 1082, IGBP 195, IGBP 298, IGBP 1035, IGBP 1160, IGBP 1051, IGBP 198, IGBP 1184
4	Cluster IV	Green	IGBP 1129, IGBP 1122, IGBP 1084, IGBP 1083, IGBP 305, IGBP 1133, IGBP 52, IGBP 1038, IGBP 1031, IGBP 61, IGBP 1149, IGBP 1056, IGBP 1173, IGBP 1040, ICMP 100697, IGBP 1148, IGBP 1050
5	Cluster V	Golden	ICMB269, Giant Bajra (C3), BAIF Bajra-1 (C4), Nutrifeed(C1), Rajka Bajri(C2)

### 3.5 Molecular characterization of pearl millet inbreds

**Figure 5:**
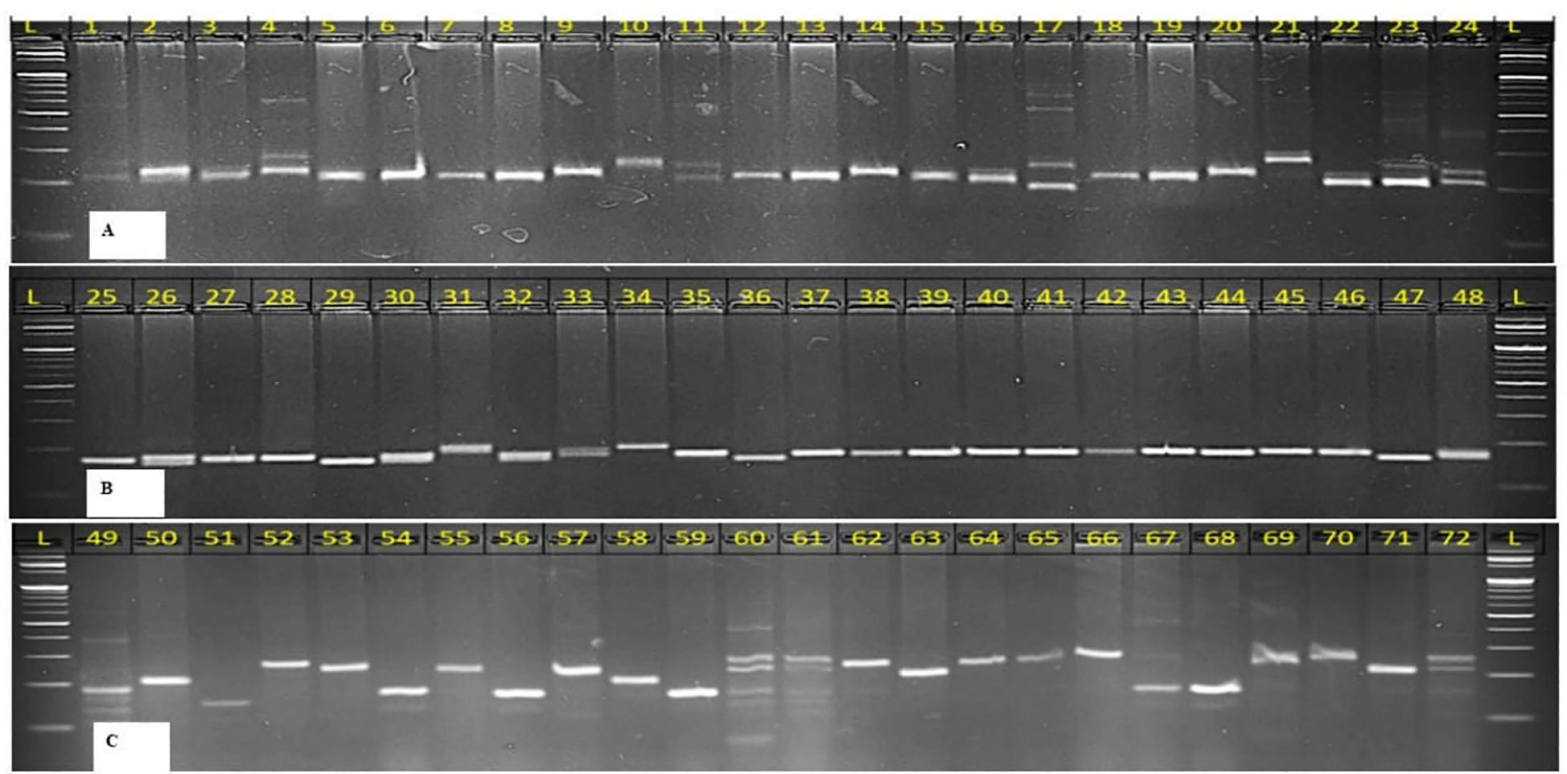
Banding profile of pearl millet germplasm using **(A)** PSMP 2251, **(B)** PSMP2263, and **(C)** PSMP2066 where, L is 100 bp ladder.

**Figure 6:**
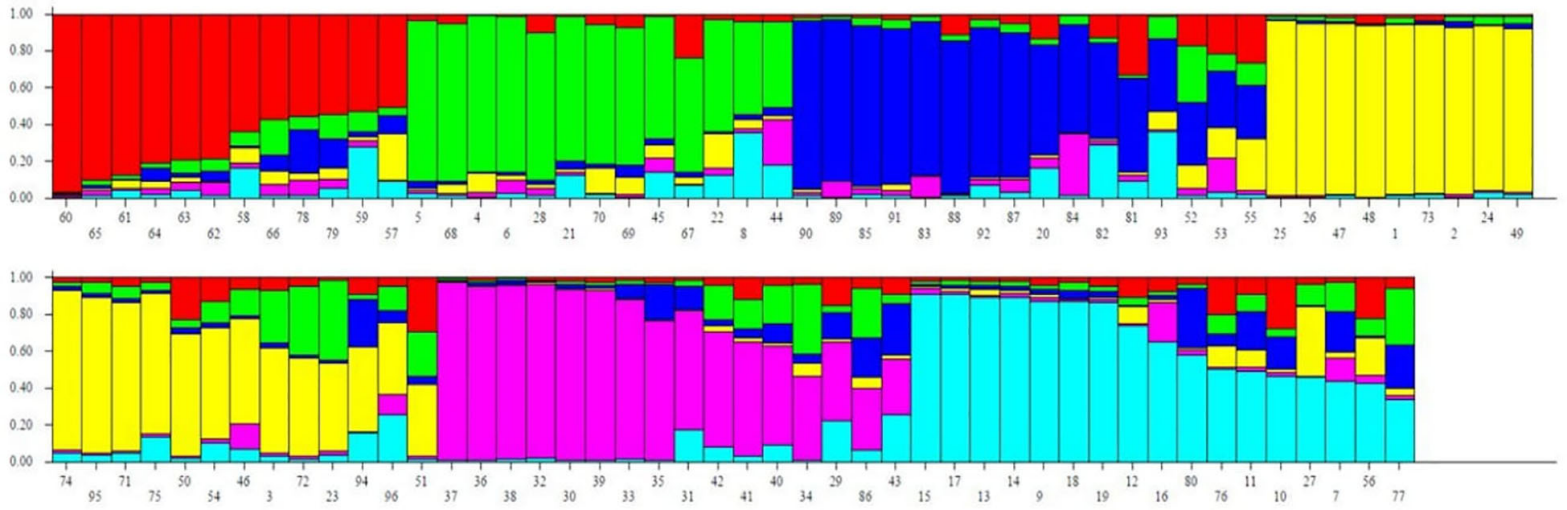
Bayesian individual clustering (A) in 96 pearl millet genotypes.

**Figure 7:**
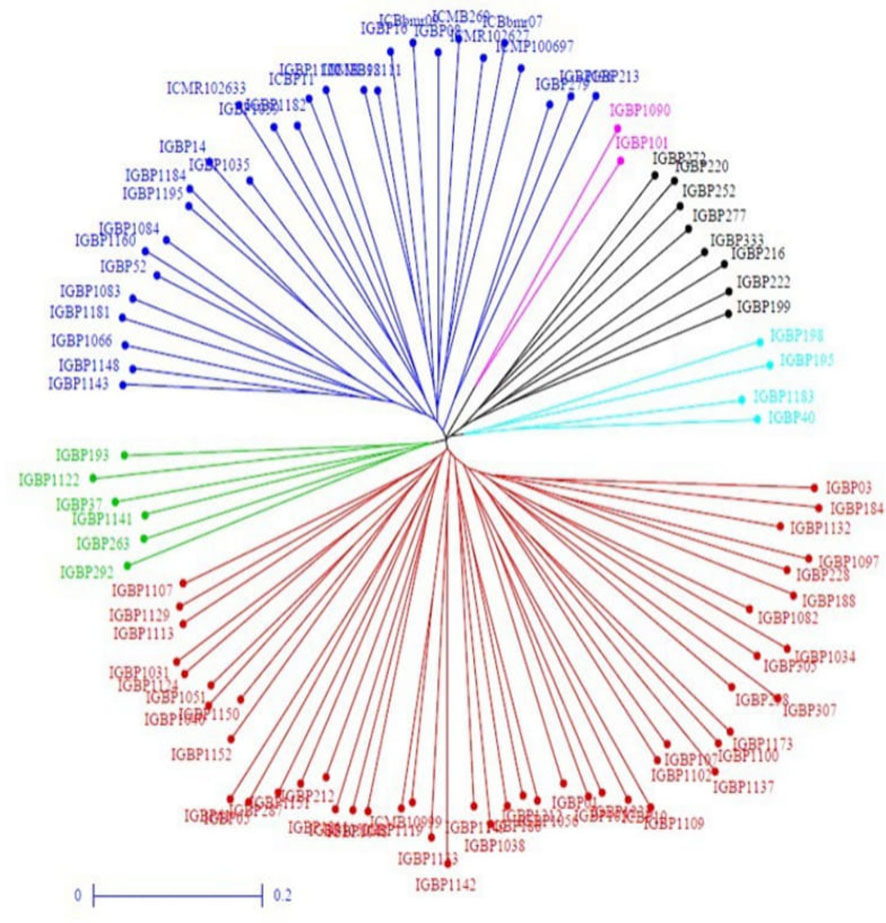
Clustering based on UPGMA (unweighted pair group method with arithmetic mean) method in 96 pearl millet genotypes by using 46 SSR primers.

**Figure 8:**
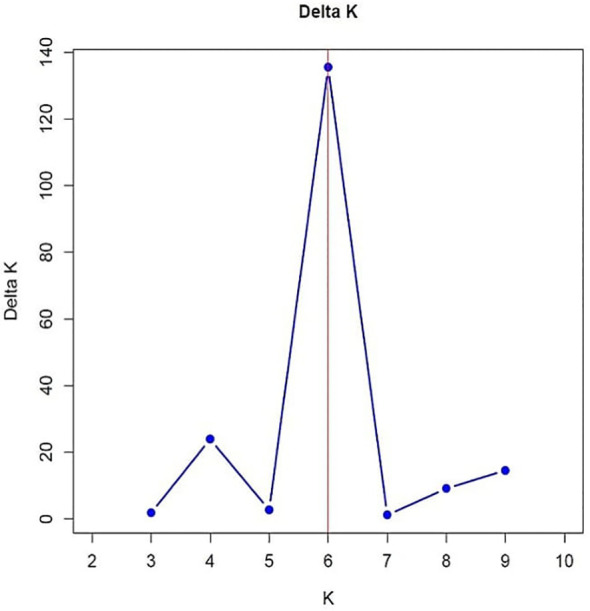
Estimated delta K values.

Analysis of Molecular Variance (AMOVA) revealed that 8% of genetic variation was attributed to differences between populations, while 80% occurred among individuals, indicating extensive gene flow and weak population structure. An additional 12% of variation was due to heterozygosity within individuals (**Table 4**). Out of 60 SSR markers screened, 46 were polymorphic and generated 203 alleles across 96 pearl millet inbreds, with an average of 5.28 alleles per locus (**Figure 5**). Gene diversity ranged from 0.28 to 0.85 (mean 0.66) and low heterozygosity (mean 0.06) reflected the inbred nature of the panel (**Supplementary Table S6**). Major allele frequency averaged 0.45, indicating substantial allelic diversity. Clustering and population structure analyses consistently resolved the genotypes into six genetic groups with varying degrees of admixture, validated by Delta K reflecting their diverse genetic backgrounds (**Figures 6**–**8**; **Supplementary Table S5**). The absence of sharply defined subpopulations suggests historical recombination and gene flow, which is advantageous for exploiting heterosis and maintaining long-term genetic gain.

**Table 4:** Analysis of molecular variance of pearl millet Inbreds based on SSR markers.

Source	df	SS	MS	Est. Var.	%
Among Populations	5	1395.01	279.00	5.21	8%
Among Individuals	90	10221.95	113.58	52.95	80%
Within Individuals	96	737.00	7.68	7.67	12%
Total	191	12353.96		65.84	100%

Further, integrating clustering analysis between morphological and molecular diversity analysis has identified 39 inbreds which are placed on common clusters (**Table 5**). They showed good agreement between morphological and molecular groupings in Groups 1, 2, and 3, indicating stable genetic relationships supported by phenotypic traits. In contrast, the remaining groups exhibited partial to complete discordance, suggesting environmental influence on morphology. Group 1 was the largest cluster (17 genotypes), reflecting broad genetic similarity, while smaller clusters represented more distinct genetic groups. Overall, the results emphasize the complementary value of combining morphological and molecular analyses for reliable genotype classification and selection.

**Table 5:** Genotype groups common in both morphological and molecular clustering.

Group	Genotypes	Morphological Cluster	Molecular Cluster	Total inbreds
1	IGBP184, IGBP01, IGBP307, IGBP1102, IGBP1057, IGBP1151, ICMB10999, IGBP1107, IGBP212, IGBP287, IGBP1142, IGBP1137, IGBP1212, IGBP1152, IGBP1119, IGBP1034, IGBP107	I	I	17
2	IGBP292, IGBP263, IGBP1141, IGBP193	II	II	4
3	IGBP1182, IGBP1195, IGBP14, IGBP1035, IGBP1160, IGBP1184	III	III	6
4	IGBP272, IGBP216, IGBP222, IGBP199	I	V	4
5	IGBP252, IGBP333	II	V	2
6	IGBP1083, IGBP1084	IV	III	2
7	IGBP198, IGBP195	III	VI	2
**8**	IGBP1183, IGBP40	I	VI	2

### 3.6 Performance of developed composites for fodder yield

Phenotypic and molecular analyses were jointly used to refine parent selection. Genotypes showing superior fodder-related traits and belonging to genetically divergent morphological and molecular clusters were prioritized. Rather than selecting extremes from a single analysis, emphasis was placed on identifying genotypes combining high biomass potential, favorable trait associations, and genetic divergence. This integrative approach ensured that selected parents represented both functional superiority and broad genetic base, strengthening the foundation for composite development. Based on phenotypic observation diverse inbred lines having synchronized flowering time were selected among different clusters developed based on morphological and molecular analysis were combined through random mating in isolation to develop composites. The days to 50% flowering of inbreds constituting different composite groups ranged as follows: IGBC-1 (62–64 days), IGBC-2 (66–67 days), IGBC-3 (65–67 days), IGBC-4 (63–65 days), IGBC-5 (61–63 days), IGBC-6 (56–59 days), IGBC-7 (58–60 days), and IGBC-8 (62–64 days). Among the eight composites developed, two composites IGBC-2 and IGBC-5 showed superior performance, recording higher green forage yields than the best check cultivar by 19.75% and 17.14% respectively (**Table 6**). These two composites will be further improved through mass selection coupled with random mating under standard isolation for one or two seasons before submitting to varietal trial.

**Table 6:** Evaluation of composites for fodder yield and its attributes.

Sl.No.	Genotype	GFY q/ha	DMY q/ha	PH (cm)	DFF	Superiority over best check for GFY
1	IGBC-1	575.82	145.57	278.57	58	5.09
2	IGBC-2	656.20	156.20	287.33	65	**19.75**
3	IGBC-3	554.63	140.63	236.40	61	1.22
4	IGBC-4	579.78	147.87	270.03	61	5.81
5	IGBC-5	641.87	150.87	278.50	63	**17.14**
6	IGBC-6	530.00	117.23	251.57	58	-3.27
7	IGBC-7	563.00	134.67	249.67	68	2.75
8	IGBC-8	576.33	144.37	246.60	59	5.18
9	C1: Giant Bajra	469.34	103.80	250.27	61	
10	C2: RBB1	461.73	103.10	240.33	58	
11	C3: BAIF Bajra 1	547.93	134.33	255.73	61	

## 4 Discussion

### 4.1 Genetic variability and trait associations governing fodder yield among inbreds

The substantial phenotypic variation observed among forage pearl millet inbreds confirms the presence of a broad genetic base ideal for biomass improvement. Importantly, the consistency of genotypic performance across rainy and summer seasons indicates that the detected variation is largely genetic rather than environment-specific, strengthening the reliability of selection decisions. This stability is particularly relevant for forage pearl millet, which is increasingly cultivated across diverse seasonal environments.

Yield-related traits such as green and dry fodder yield exhibited high heritability coupled with large phenotypic and genotypic variation, suggesting that selection for biomass can be highly effective even in early-generation inbred material (Abubakar et al., 2019; Singh et al., 2014). Root traits consistently exhibited high heritability and genetic advance, emphasizing their importance for enhancing resource-use efficiency, drought resilience, and regrowth potential—key attributes for forage productivity.

### 4.2 Role of shoot–root coordination and physiological traits in biomass accumulation

The correlation analysis provides clear insight into how multiple traits regulate fodder yield. Strong positive associations of green fodder yield with stem girth, plant height, root projected area, root diameter, and root volume indicate that biomass accumulation in pearl millet is governed by coordinated shoot vigor and a robust root system. These relationships emphasize that selection solely based on above-ground biomass may overlook critical below-ground contributors to yield stability and resilience. Similar patterns reported in previous studies (Burman et al., 2011; Bind et al., 2015).

The negative association between canopy temperature and fodder yield further suggests that physiological efficiency and stress avoidance play an important role in biomass production. Genotypes maintaining cooler canopies likely benefit from improved transpiration efficiency and water status, translating into greater dry matter accumulation (Gong et al., 2023). Collectively, these findings support an integrated selection strategy that combines morphological, root, and physiological traits rather than relying on yield alone.

### 4.3 Multivariate analyses

The PCA–correlation analysis emphasized root architectural traits as primary drivers of genetic divergence, consistent with their recognized role in enhancing resource acquisition, biomass production, and stress tolerance (Jia et al., 2019). The independent contribution of shoot, physiological, and yield-related traits along PC2 indicates a complementary mechanism governing productivity. The inbreds such as IGBP212, IGBP122, IGBP1043, IGBP287, and IGBP16 combined favorable root and yield traits, aligning with the concept of ideotype-based selection for complex traits (Donald, 1968). The clustering of several genotypes near the origin reflects phenotypic stability but limited discriminatory potential, a pattern commonly observed in PCA studies of diverse germplasm (Hair et al., 2019). Overall, these results validate the use of PCA coupled with correlation analysis as an effective tool for identifying key traits and elite genotypes for breeding programs targeting productivity and stress adaptation (Arya et al., 2018; Sharma et al., 2025, Arvinth et al., 2024) identifying root traits, yield components, and physiological parameters as key discriminators in pearl millet diversity.

Cluster analysis further enabled the identification of genetically divergent genotype groups with contrasting trait profiles. Large inter-cluster distances, particularly between clusters containing high-yielding and low-yielding genotypes, indicate substantial scope for recombining complementary traits. Tesfaye, 2022 reported parental selection should be done between clusters for hybridization. These results demonstrate that multivariate tools are essential for moving beyond trait-by-trait selection and for identifying genotypes that combine multiple desirable attributes. We have selected inbred lines between these diverse cluster and developed composites. The evaluation of these composites successfully showed higher yield compared to check cultivars. These findings support the use of cluster-based selection to enhance genetic gain and guide parent selection in forage pearl millet improvement programs.

### 4.4 Molecular diversity complements phenotypic selection

SSR-based molecular analysis revealed high allelic diversity and weak population structure, with most genetic variation residing among individuals rather than between populations (Jorben et al., 2024; Ambawat et al., 2025; Babu et al., 2024; Gunguniya et al., 2023). Such patterns are typical of outcrossing species like pearl millet and are advantageous for breeding programs aiming to exploit heterosis and maintain long-term genetic gain. Such patterns suggest extensive gene flow and align with earlier findings (Ramakrishnan et al., 2016; Panwar et al., 2010; Gopal et al., 2023), reinforcing the presence of broad genetic differentiation within the germplasm. This diversity is advantageous for breeding programs seeking to exploit heterosis and develop high-performing, stress-resilient cultivars.

Heterosis in pearl millet can be effectively exploited through the development of both hybrids and composites (Yadav et al., 2021). The superior performance of selected composites validates the integrative selection strategy employed in this study. By combining inbreds chosen for high biomass-related traits, synchronized flowering, favorable multivariate positioning, and genetic divergence, the resulting composites captured complementary alleles contributing to enhanced fodder yield. The observed yield advantage over commercial checks demonstrates that composites remain a practical and effective approach for forage pearl millet improvement, particularly where hybrid seed production may be less feasible.

These findings highlight that systematic integration of phenotypic, multivariate, and molecular information can accelerate the development of improved forage cultivars and help break existing yield plateaus in fodder pearl millet.

The superior composites identified will undergo further improvement and stabilization through one or two cycles of random mating under isolation, accompanied by negative mass selection to eliminate undesirable phenotypes. Upon stabilization, these composites will be submitted to the ICAR–AICRP on Forage Crops and Utilization for multilocation varietal trials, paving the way for their potential release as high-performing forage cultivars.

## 5 Conclusions

This study demonstrated that the systematic integration of multi-trait phenotypic evaluation with SSR-based molecular diversity analysis is an effective strategy for developing superior fodder pearl millet composites. Significant variation was observed for key traits including plant height, leaf dimensions, fodder yield, chlorophyll content, total root length, and root diameter. Morphological and SSR based clustering grouped the genotypes into distinct clusters, the diversity information was strategically exploited to select complementary inbreds possessing high biomass potential, desirable regrowth ability, and favourable phenological traits. These selected inbreds were intermated to develop composite populations, which were subsequently evaluated for fodder yield performance. The superior performance of specific composites over standard check cultivars confirms selection of genetically diverse inbreds based on phenotype and molecular diversity can effectively enhance the fodder yield. Overall, the study provides a practical framework for utilizing genetic diversity to develop high-biomass fodder pearl millet composites and supports their potential use in future forage improvement programs.

## Data Availability

The original contributions presented in the study are included in the article/**Supplementary Material**. Further inquiries can be directed to the corresponding authors.
