# Supplementary material for: Integrating morphological and molecular diversity to develop high-biomass fodder pearl millet composites
Source: Front Plant Sci. 2026 Feb 18;17:1767075. doi: 10.3389/fpls.2026.1767075 (PMC12957238; doi:10.3389/fpls.2026.1767075)
Supplement: Supplementary file 1 [file Table1.docx]

**Supplementary Table 1: List of pearl millet inbreds and their pedigrees**

| **S. No.** | Genotype | **Accession** | **Year of development** | **Country of origin** |
| --- | --- | --- | --- | --- |
| 1 | IGBP1035 | IP 7633-S1-S5-S6 | 2021-2024 | India |
| 2 | IGBP1148 | IP 22419-S1-S5-S6-S8 | 2020-2024 | India |
| 3 | IGBP1059 | IP 3098-S1-S5-S6 | 2021-2024 | India |
| 4 | IGBP1143 | IP 18434-S1-S5-S6 | 2021-2024 | India |
| 5 | IGBP1084 | IP 10427-S1-S5-S6-S7 | 2021-2024 | India |
| 6 | IGBP1122 | IP 3732-S1-S5-S6 | 2021-2024 | India |
| 7 | IGBP1083 | IP 2269-S1-S5-S6-S7 | 2021-2024 | India |
| 8 | IGBP1066 | (IP 14506 x DRSB2)-S1-S5-S6-S7 | 2020-2024 | India |
| 9 | IGBP1160 | IP 10471-S1-S5-S6 | 2021-2024 | Zimbabwe |
| 10 | IGBP52 | Giant bajra-S1--S5-S6-S8 | 2020-2024 | India |
| 11 | IGBP1181 | IP 5816-S1-S5-S6 | 2021-2024 | Senegal |
| 12 | IGBP1184 | IP 18090-S1-S5-S6 | 2021-2024 | India |
| 13 | IGBP1195 | IP 5031-S1-S5-S6 | 2021-2024 | Nigeria |
| 14 | IGBP37 | Giant bajra-S1-S5-S6-S8 | 2020-2024 | India |
| 15 | IGBP1182 | IP 8069-S1-S5-S6 | 2021-2024 | India |
| 16 | IGBP08 | IC384998-S1-S5-S6-S8 | 2020-2024 | India |
| 17 | IGBP184 | ICMFA1605-S1-S5-S6-S8 | 2020-2024 | India |
| 18 | IGBP228 | PPM1084-S1-S5-S6 | 2020-2024 | India |
| 19 | 1GBP186 | IC384990-S1-S5-S6-S8 | 2020-2024 | India |
| 20 | IGBP298 | PT6686-S1-S5-S6-S8 | 2020-2024 | India |
| 21 | IGBP305 | Prathap-S1-S5-S6-S8 | 2020-2024 | India |
| 22 | IGBP307 | TSFB15-8-S1-S5-S6-S8 | 2020-2024 | India |
| 23 | IGBP1097 | Rajasthan landrace-S1-S5-S6-S7 | 2020-2024 | India |
| 24 | IGBP1141 | IP 9824-S1-S5-S6 | 2021-2024 | Mozambique |
| 25 | IGBP1137 | IP 13384-S1-S5-S6 | 2021-2024 | Uganda |
| 26 | IGBP1034 | IP 20348-S1-S5-S6 | 2021-2024 | India |
| 27 | IGBP01 | Moti bajra-S1-S5-S6-S8 | 2020-2024 | India |
| 28 | IGBP1082 | IP 8761-S1-S5-S6 | 2020-2024 | Botswana |
| 29 | IGBP1057 | IP 17554-S1-S5-S6 | 2021-2024 | Togo |
| 30 | IGBP1223 | IP 4378-S1-S5-S6 | 2021-2024 | India |
| 31 | IGBP03 | IC385007-S1-S5-S6-S8 | 2020-2024 | India |
| 32 | IGBP1109 | IP 8002-S1-S5-S6 | 2021-2024 | Sudan |
| 33 | IGBP1100 | IP 3122-S1-S5-S6 | 2021-2024 | India |
| 34 | IGBP1173 | IP 10761-S1-S5-S6 | 2021-2024 | Sudan |
| 35 | IGBP1102 | IP 10953-S1-S5-S6 | 2021-2024 | Kenya |
| 36 | IGBP107 | PT6686-S1-S5-S6-S8 | 2020-2024 | India |
| 37 | IGBP1132 | IP 9406-S1-S5-S6 | 2021-2024 | Ghana |
| 38 | IGBP1090 | IP 15533-S1-S5-S6 | 2021-2024 | Burkina Faso |
| 39 | IGBP196 | Moti bajra-S1-S5-S6-S8 | 2020-2024 | India |
| 40 | IGBP199 | Moti bajra-S1-S6-S7-S8 | 2020-2024 | India |
| 41 | IGBP212 | ICMFA1602A4-S1-S5-S6 | 2020-2024 | India |
| 42 | IGBP213 | IC384986-S1-S5-S6-S8 | 2020-2024 | India |
| 43 | IGBP216 | AVKB19-S1-S5-S6-S8 | 2020-2024 | India |
| 44 | IGBP220 | AVKB7-S1-S5-S6-S8 | 2020-2024 | India |
| 45 | IGBP222 | IC384987-S1-S5-S6-S8 | 2020-2024 | India |
| 46 | IGBP252 | IC384990-S1-S5-S6-S8 | 2020-2024 | India |
| 47 | IGBP263 | ICMATS111-S1-S5-S6 | 2021-2024 | India |
| 48 | IGBP333 | PT6686-S1-S5-S6-S8 | 2020-2024 | India |
| 49 | IGBP272 | Giant bajra-S1-S4-S5-S8 | 2020-2024 | India |
| 50 | IGBP277 | Giant bajra-S1-S5-S6-S8 | 2020-2024 | India |
| 51 | IGBP1133 | IP 7470-S1-S5-S6 | 2021-2024 | Tanzania |
| 52 | IGBP1212 | IP 7536-S1-S5-S6 | 2021-2024 | India |
| 53 | IGBP1107 | GFB 1-S1-S5-S6-S7 | 2020-2024 | India |
| 54 | IGBP1183 | IP 7470-S1-S5-S6 | 2021-2024 | Tanzania |
| 55 | IGBP1120 | IP 7910-S1-S5-S6 | 2021-2024 | Niger |
| 56 | IGBP1056 | IP 6802-S1-S5-S6-S8 | 2020-2024 | India |
| 57 | IGBP1050 | IP 20440-S1-S5-S6-S8 | 2020-2024 | India |
| 58 | IGBP1048 | IP 11336-S1-S5-S6-S8 | 2020-2024 | India |
| 59 | IGBP1051 | AVKB 9-S1-S5-S6-S8 | 2020-2024 | Togo |
| 60 | IGBP1040 | RBB1-1-P1-S1-S5-S6-S8 | 2020-2024 | India |
| 61 | IGBP1031 | IP 3757-S1-S5-S6 | 2021-2024 | India |
| 62 | IGBP1124 | IP 21517-S1-S5-S6 | 2021-2024 | Niger |
| 63 | IGBP1129 | IP 4974-S1-S5-S6 | 2021-2024 | Nigeria |
| 64 | IGBP1150 | IP 13180-S1-S5-S6 | 2021-2024 | Nigeria |
| 65 | IGBP1152 | IP 8074-S1-S5-S6 | 2021-2024 | India |
| 66 | IGBP1119 | IPC 804-S1-S5-S6 | 2021-2024 | India |
| 67 | ICMB10999 | ICMB10999-S1-S5-S6 | 2021-2024 | India |
| 68 | IGBP188 | ICMV1601-S1-S5-S6 | 2021-2024 | India |
| 69 | IGBP193 | IC384986-S1-S5-S6-S8 | 2020-2024 | India |
| 70 | IGBP195 | IC389981-S1-S5-S6-S8 | 2020-2024 | India |
| 71 | IGBP198 | Raj local-S1-S5-S6-S8 | 2020-2024 | India |
| 72 | IGBP287 | TSFB15-8-S1-S5-S6-S8 | 2020-2024 | India |
| 73 | IGBP292 | Giant bajra x AVKB-34-S1-S5-S6-S7 | 2020-2024 | India |
| 74 | IGBP1142 | IP 9532-S1-S5-S6 | 2021-2024 | Ghana |
| 75 | IGBP14 | IC385006-S1-S5-S6-S7 | 2020-2024 | India |
| 76 | IGBP40 | Giant bajra -S1-S6-S7 | 2020-2024 | India |
| 77 | IGBP1151 | IP 16402-S1-S5-S6 | 2021-2024 | Zimbabwe |
| 78 | IGBP61 | Giant bajra x AVKB-34-S1-S6-S7 | 2020-2024 |  |
| 79 | IGBP05 | IP11336-S1-S5-S6 | 2020-2024 | Burkina Faso |
| 80 | ICBP19 | IC384993-S1-S5-S6-S8 | 2020-2024 | India |
| 81 | IGBP279 | PT6686-S1-S5-S7 | 2020-2024 | India |
| 82 | IGBP1149 | IP 18293-P152-S1-S5-S6 | 2020-2024 | India |
| 83 | IGBP1038 | IP8642-S1-S5-S6-S8 | 2020-2024 | India |
| 84 | IGBP1211 | IP 15512-S1-S5-S6 | 2021-2024 | Burkina Faso |
| 85 | IGBP1113 | IP 6869-S1-S5-S6 | 2021-2024 | Kenya |
| 86 | ICBP11 | ICBP 83-S1-S5-S6 | 2021-2024 | India |
| 87 | ICMB269 | B-comp HS-27-3-3-3-5-B-S1-S5-S6 | 2020-2024 | India |
| 88 | ICMR102633 | ICMR102633-S1-S5-S6 | 2021-2024 | India |
| 89 | ICMP100697 | ICMP100697-S1-S5-S6 | 2021-2024 | India |
| 90 | ICBbmr07 | ICMV 167005-S1-S5-S6 | 2020-2024 | India |
| 91 | ICMR102627 | IP 6140 S1-3-1-2-4-4-B-B-B-B-B | 2020-2024 | India |
| 92 | ICMB12111 | IP 2761 S1-9-4-2-2-2-3-5-1-B-B | 2020-2024 | India |
| 93 | ICMB298 | [(ICMB 95444 x ICMB 93333)-24-2-B-2-BX(BSECBPT/91-38 x SPF3/S91-529)-10-1-7-16]-18-5-B-S1-S5-S6 | 2020-2024 | India |
| 94 | ICBbmr09 | WRPbmrS1-19-7-3-3-2-2-1-B-B | 2021-2024 | India |
| 95 | IGBP16 | IC384993-S1-S5-S6-S8 | 2020-2024 | India |
| 96 | IGBP101 | PT6686-S1-S6-S7 | 2020-2024 | India |

## **Supplementary table 2: List of SSR markers used for genetic diversity analysis**

| **S. No.** | **Primer Name** | **Forward primer sequence** | **Reverse primer sequence** |
| --- | --- | --- | --- |
| **1** | **CTM21** | ATGCCTCCCACCCCACGTCG | CGTCGCACTAGCCACAGTCA |
| **2** | **CTM25** | GCGAAGTAGAACACCGCGCT | GCACTTCCTCCTCGCCGTCA |
| **3** | **CTM-9** | GCCTCCTCTTGATACCATATT | TAGCCTTGGCTGCTATATTC |
| **4** | **CUMP009** | ATCTGATCGTGAGGCCTCAAC | GCCGACCAAGAACTTCATACAAT |
| **5** | **CUMP010** | GCTGAACTATTCTGTAAACTTAAC | TATCGAAACGGTACTAAAATCATG |
| **6** | **IPES0236** | GGCCAGCTCGCCTAGAT | AGATCCACCGCCTAATGAAA |
| **7** | **PGIRDI9** | TGAGGACCGAGAAGAAGCAT | CAACACCCAACAGAAACTGAA |
| **8** | **IPES0007** | ACACCTCGCTGCACCTCTA | GCAACACAGATGAGACTGGC |
| **9** | **IPES0013** | CCTCTGGCAGTGGTCGTAGT | GAACTGAGGTAGAACCCCGC |
| **10** | **IPES0022** | GGAACACATACGGAGTGACAGA | TGTGTCTTACCCCTTGCTGA |
| **11** | **IPES0052** | GGATCATCGATCAGCCGAC | CATGGTGATCTGAATCGCAG |
| **12** | **IPES0066** | CAACATGTCAAGGAAGTAAAATTGA | GCCTCTTGATACCCAAGATCA |
| **13** | **IPES0068** | AGTCCGGAAGAGGAGACCAC | TGGTGGTGATCTGATGCCTA |
| **14** | **IPES0098** | ATCAAGCTTCATACCCCTGC | CATCTTCTTCATCATCTTTCGC |
| **15** | **IPES0102** | ACATGTGTTGGCTTGCTGTG | CATCCTGTCCTGTCGTGCTA |
| **16** | **IPES0118** | AAGGTGCAGAAGTTCACGCT | TTTTACAATCACGGCACGAC |
| **17** | **IPES0145** | TCTTGGGATCCGATGATGA | ACAAAGCCACAGCACAACAG |
| **18** | **IPES0146** | CATCAGAATACGGACGCCTT | CATCAGCTTTGGAGTCAGCA |
| **19** | **IPES0163** | AAGATCAAGGCCAGCAACTG | GAGAGTGCACCTGTGCAAAA |
| **20** | **IPES0185** | TCTGCTTGTGTTTTACCCCC | CGTTGGTACCCATGATTTTCA |
| **21** | **IPES0198** | GGGGAGCTCTCTCTGAACTG | GAACCGCTTCTTCATCCATC |
| **22** | **IPES0208** | CGAAGGAGGAGTACGACGAG | TCCACAAGGTGACCTCACTG |
| **23** | **PGIRD50** | CTCTCGGTTTGACGGTTTGT | GGGGAAAACAAAGTTGCTCA |
| **24** | **PGIRD57** | GGCCCCAAGTAACTTCCCTA | TCAAGCTAGGGCCAATGTCT |
| **25** | **PSMP2001** | CATGAAGCCAATTAGGTCTC | ACCATCTGACTTGTTCTTATCC |
| **26** | **PSMP2006** | GACTTATAGTCACTGGGAAAGCTC | GCTTTAATAACTTTGTGCGTATT |
| **27** | **PSMP2008** | GATCATGTTGTCATGAATCACC | ACACTACACCTACATACGCTCC |
| **28** | **PSMP2018** | CGCAAGACATTTTAGTATCACC | ACAGTCATCCTCAGTCGTCC |
| **29** | **PSMP2030** | ACCAGAGCTTGGAAATCAGCAC | CATAATGCTTTCAAATCTGCCACAC |
| **30** | **PSMP2043** | TCATATTCTCCTGTCTAAAACGTC | ACAAATCGTACAAGTTCCACTC |
| **31** | **PSMP2063** | GAGCACATGAAATAGGAAGCAG | AAGGTAGTTATAGTTAGCTTGATC |
| **32** | **PSMP2066** | ATATTAGAGCATTGCATCGC | GCATACCAGCATACAGCAGCAA |
| **33** | **PSMP2068** | CAATAACCAAACAAGCAGGCAG | CTTCACTCCCACCCTTTCTAATTC |
| **34** | **PSMP2251** | AAAGTGAATACGATACAGGAGCTGAG | CATTTCAGCCGTTAAGTGAGACAA |
| **35** | **PSMP2253** | CAGGTGATCTGTCTGGTTTCCTAATC | TAGCCACTGGAGTGCTACTGAA |
| **36** | **PSMP2255** | CATCTAAACACAACCAATCTTGAAC | TGGCACTCTTAAATTGACGCAT |
| **37** | **PSMP2263** | AACCCCACCAGTAAGTTGTGCTGC | GATGACGACAAGACCTTCTCTCC |
| **38** | **PSMP2074** | AGGACTGTAGGAGTGTGGACAA | CCAGACCTACCAGTGAATGAGA |
| **39** | **PSMP2076** | GGAATAGTTATTGGCAAAATGTG | ATACTACACACTGTAAGCATTG |
| **40** | **PSMP2077** | GCCAAATTATTCCCAAGTGAACA | CTCTTGGTTGCATATCTTTCTTTT |
| **41** | **PSMP2078** | CATGCCCATGACAGTATCTTAAT | ACTGTTTCGGTTCCAAAATACTT |
| **42** | **PSMP2086** | CGCTTGTTTTCCTTTCTTCTTGTT | CCTTCTCAGATCCTGTGCTTTCTT |
| **43** | **PSMP2088** | AAGAAGCCACCAGCACAAAA | TGCATGAAAGTAGAGGATGGTAAA |
| **44** | **PSMP2201** | CCCGACGTTATGCGTTAAGTT | TCCATCCATCCATTAATCCACA |
| **45** | **PSMP2202** | CTGCCTGTTGAGAATAAATGAG | GTTCCGAATATAGAGCCCAAG |
| **46** | **PSMP2203** | GAACTTGATGAGTGCCACTAGC | TTGTGTAGGGAGCAACCTTGAT |
| **47** | **PSMP2206** | AGAAGAAGAGGGGGTAAGAAGGAG | AGCAACATCCGTAGAGGTAGAAG |
| **48** | **PSMP2207** | CAGGGCATACTTCAAGATTG ATTC | GTCCACTIGTTATICTCTATCACC |
| **49** | **PSMP2225** | CCGTACTGATGATACTGATGGTT | TGGGAGGTAAGCTCAGTAGTGT |
| **50** | **PSMP2229** | CCACTACCATCGTCTTCCTCCATTC | GTCCGTTCCGTTAGTTGTTGCC |
| **51** | **PSMP2211** | CTGCATGACGTGTGACCAATACC | AACAAATCAGCACCAGCCTCC |
| **52** | **PSMP2050** | TCAAACGGCATCAGACAACAAC | GGATCTCTTAGTGTGGTGGAGAGC |
| **53** | **CTM1** | TCTGGGGATTGGCTGGAATTACA | AAGTTGGGTAACGCCAGGGTTTTC |
| **54** | **CTM27** | GTTGCAAGCAGGAGTAGATCGA | CGCTCTGTAGGTTGAACTCCTT |
| **55** | **PSMP2059** | GGGGAGATGAGAAAACACAATCAC | TCGAGAGAGGAACCTGATCCTAA |
| **56** | **PSMP2069** | ACAGAAAAAGAGAGGCACAGGAGA | GCCACTCGATGGAAATGTGAAATC |
| **57** | **PSMP2086** | CGCTTGTTTTCCGGGCITGCTGTT | CGCTTGTTTTCCTITCTTGCTGTT |
| **58** | **PSMP2214** | CGCACAGTACGTGTGAGTGAAG | GATTGAGCAGCAAAAACCAGC |
| **59** | **PSMP2235** | GCTTTTCTGCTTCTCCGTAGAC | CCCAACAATAGCCACCAATAAAGA |
| **60** | **SiNAS-2** | GCAACTACCTCGACCTGAGC | GTCCACGACGGGGTACAG |

**Supplementary Table 3: REML-Based Analysis of Variance (ANOVA) for Morphological, Phenological, Yield, Physiological, and Root Traits Across Genotypes**

|  | **Parameters** | **Genotypes** | **Residuals** |
| --- | --- | --- | --- |
|  | **d.f** | 99 | 290 |
| **Mean Sum of Square** | **Plant Height** | 1758.60** | 653.13 |
|  | **Leaf length** | 161.41** | 60.21 |
|  | **Leaf width** | 0.84** | 0.48 |
|  | **Flag leaf area** | 5701.20** | 136.30 |
|  | **Leaf area** | 4985.90** | 622.60 |
|  | **Spike Length** | 71.72** | 6.27 |
|  | **Spike Girth** | 67.30** | 4.40 |
|  | **Days to 50% flowering** | 39.78** | 3.02 |
|  | **Days to Maturity** | 5.26 | 26.57 |
|  | **Productive tillers/pt** | 16.43** | 0.63 |
|  | **Stem Girth** | 9.09** | 3.25 |
|  | **Leaf to Stem Ratio** | 0.07 | 0.05 |
|  | **Nodes/plant** | 4.28** | 2.65 |
|  | **Green Fodder Yield** | 69.11** | 1.16 |
|  | **Dry Fodder Yield** | 2.78** | 0.03 |
|  | **Regeneration 1%** | 905.91** | 263.58 |
|  | **Regeneration 2%** | 738.73** | 130.68 |
|  | **Total Chlorophyll Content** | 256.70** | 9.05 |
|  | **Canopy Temperature** | 1.14 . | 0.91 |
|  | **Total Soluble Solids** | 4.64** | 0.09 |
|  | **Root Projected Area** | 843.95** | 5.55 |
|  | **Total Root Length** | 25641.00** | 199.60 |
|  | **Root Tips** | 75103** | 683 |
|  | **Forks** | 60902.00** | 143.00 |
|  | **Segments** | 337147** | 4362 |
|  | **Maximum Root Diameter** | 1.20** | 0.01 |
|  | **Average Root Diameter** | 0.05** | 0 |
|  | **Estimated Root Volume** | 1166.77** | 140.13 |
|  | **Primary Root Length** | 102.39** | 2.12 |

Significance. Codes: ** 0.01, * 0.05

## **Supplementary table 4. The combination of inbreds used in development of composites**

| **Sl. No.** | **Name of composites** | **Inbreds** |
| --- | --- | --- |
| **1** | IGBC-1 | ICMB 12111, IGBP 1109, IGBP40, ICMR102627, IGBP 1182, IGBP 1173 |
| **2** | IGBC-2 | ICMR 102633, IGBP292, IGBP1043, IGBP 1160, IGBP333, IGBP 1102, IGBP 216, |
| **3** | IGBC-3 | IGBP184, IGBP 263, IGBP279, IGBP 1082, IGBP252, |
| **4** | IGBC-4 | ICMB10999, IGBP1141, IGBP196, IGBP216, IGBP 1173, IGBP 1050 |
| **5** | IGBC-5 | IGBP 1107, IGBP1102, ICMR102633 IGBP222, IGBP 1183, IGBP 1050 |
| **6** | IGBC-6 | IGBP1031, IGBP 333, IGBP220, IGBP252, IGBP 1173 |
| **7** | IGBC-7 | IGBP1056, IGBP 1142, ICMB269, IGBP1090, IGBP 199 |
| **8** | IGBC-8 | IGBP 37, IGBP287, IGBP16, IGBP 1090, IGBP198, IGBP 1129, |

**Supplementary table 5: Distribution of 96 pearl millet genotypes into different clusters using UPGMA by Neighbor-Joining method.**

| **S.no** | **Cluster number** | **Cluster colour** | **Genotypes** |
| --- | --- | --- | --- |
| 1 | **Cluster I** | **Red** | IGBP03, IGBP184, IGBP1132, IGBP1097, IGBP228, IGBP188, IGBP1082, IGBP1034, IGBP305, IGBP307, IGBP298, IGBP1173, IGBP1100, IGBP1137, IGBP107, IGBP1102, IGBP1109, IGBP19, IGBP1223, IGBP1057, IGBP01, IGBP1056, IGBP1212, IGBP186, IGBP1038, IGBP1149, IGBP1142, IGBP1133, ICMB10999, IGBP1119, IGBP1048, IGBP1050, IGBP1211, IGBP212, IGBP1151, IGBP287, IGBP05, IGBP61, IGBP1152, IGBP1150, IGBP1040, IGBP1051, IGBP1124, IGBP1031, IGBP1113, IGBP1129, IGBP1107 |
| 2 | **Cluster II** | **Green** | IGBP292, IGBP263, IGBP1141, IGBP37, IGBP1122, IGBP193 |
| 3 | **Cluster III** | **Blue** | IGBP1143, IGBP1148, IGBP1066, IGBP1181, IGBP1083, IGBP52, IGBP1160, IGBP1084, IGBP1195, IGBP1184, IGBP14, IGBP1035, ICMR102633, IGBP1059, IGBP1182, ICBP11, IGBP1120, ICMB298, ICMB12111, IGBP16, ICBbmr09, ICBP08, ICMB269, ICMR102627, ICBbmr07, ICMP100697, IGBP279, IGBP196, IGBP213 |
| 4 | **Cluster IV** | **Pink** | IGBP1090, IGBP101 |
| 5 | **Cluster V** | **Black** | IGBP272, IGBP220, IGBP252, IGBP277, IGBP333, IGBP216, IGBP222, IGBP199 |
| 6 | **Cluster VI** | **Cyan** | IGBP198, IGBP195, IGBP1183, IGBP40 |

## **Supplementary Table 6: Genetic Diversity parameters generated using PowerMarker v3.25**

| **Marker** | **Major Allele Frequency** | **Genotype Number** | **Allele Number** | **Gene Diversity** | **Heterozygosity** | **PIC** | **Inbreeding coeffcient** |
| --- | --- | --- | --- | --- | --- | --- | --- |
| **CTM21** | 0.33 | 6.00 | 6.00 | 0.75 | 0.00 | 0.71 | 1.00 |
| **CTM25** | 0.31 | 11.00 | 8.00 | 0.78 | 0.04 | 0.75 | 0.95 |
| **CUMP009** | 0.36 | 6.00 | 5.00 | 0.70 | 0.02 | 0.65 | 0.97 |
| **PSMP2076** | 0.41 | 6.00 | 6.00 | 0.69 | 0.01 | 0.63 | 0.99 |
| **PGIRD19** | 0.41 | 8.00 | 6.00 | 0.68 | 0.13 | 0.62 | 0.82 |
| **IPES0007** | 0.52 | 5.00 | 5.00 | 0.63 | 0.01 | 0.58 | 0.98 |
| **IPES0013** | 0.51 | 8.00 | 5.00 | 0.58 | 0.07 | 0.49 | 0.88 |
| **IPES0022** | 0.46 | 4.00 | 4.00 | 0.63 | 0.00 | 0.56 | 1.00 |
| **IPES0185** | 0.68 | 8.00 | 5.00 | 0.50 | 0.08 | 0.46 | 0.83 |
| **PSMP2066** | 0.21 | 21.00 | 8.00 | 0.85 | 0.20 | 0.83 | 0.77 |
| **PSMP2078** | 0.28 | 15.00 | 7.00 | 0.81 | 0.11 | 0.79 | 0.86 |
| **PSMP2077** | 0.26 | 11.00 | 7.00 | 0.82 | 0.08 | 0.79 | 0.90 |
| **PSMP2018** | 0.26 | 20.00 | 9.00 | 0.85 | 0.23 | 0.83 | 0.73 |
| **CUMP010** | 0.67 | 4.00 | 4.00 | 0.51 | 0.00 | 0.47 | 1.00 |
| **PSMP2006** | 0.32 | 9.00 | 6.00 | 0.76 | 0.04 | 0.72 | 0.95 |
| **PSMP2043** | 0.61 | 3.00 | 3.00 | 0.00 | 0.00 | 0.49 | 1.00 |
| **PSMP2068** | 0.36 | 9.00 | 7.00 | 0.77 | 0.03 | 0.74 | 0.96 |
| **PSMP2201** | 0.66 | 3.00 | 3.00 | 0.50 | 0.00 | 0.45 | 1.00 |
| **PSMP2202** | 0.71 | 3.00 | 3.00 | 0.42 | 0.00 | 0.34 | 1.00 |
| **PSMP2255** | 0.32 | 6.00 | 6.00 | 0.73 | 0.00 | 0.69 | 1.00 |
| **PSMP2203** | 0.33 | 6.00 | 6.00 | 0.76 | 0.00 | 0.72 | 1.00 |
| **PSMP2086** | 0.71 | 5.00 | 4.00 | 0.44 | 0.21 | 0.39 | 0.53 |
| **PSMP2207** | 0.52 | 4.00 | 4.00 | 0.62 | 0.00 | 0.56 | 1.00 |
| **PSMP2225** | 0.84 | 4.00 | 3.00 | 0.28 | 0.09 | 0.27 | 0.67 |
| **IPES0145** | 0.68 | 7.00 | 4.00 | 0.50 | 0.17 | 0.47 | 0.67 |
| **PSMP2030** | 0.42 | 10.00 | 6.00 | 0.72 | 0.08 | 0.68 | 0.89 |
| **IPES0118** | 0.74 | 9.00 | 6.00 | 0.43 | 0.10 | 0.39 | 0.76 |
| **IPES0236** | 0.36 | 7.00 | 5.00 | 0.76 | 0.38 | 0.72 | 0.51 |
| **IPES0208** | 0.41 | 11.00 | 6.00 | 0.69 | 0.09 | 0.63 | 0.87 |
| **PSMP2251** | 0.29 | 11.00 | 7.00 | 0.79 | 0.10 | 0.76 | 0.87 |
| **PSMP2263** | 0.38 | 10.00 | 6.00 | 0.72 | 0.07 | 0.67 | 0.90 |
| **PSMP2074** | 0.43 | 6.00 | 4.00 | 0.67 | 0.23 | 0.61 | 0.66 |
| **PSMP2206** | 0.51 | 4.00 | 4.00 | 0.64 | 0.00 | 0.59 | 1.00 |
| **PSMP2229** | 0.41 | 8.00 | 6.00 | 0.74 | 0.07 | 0.70 | 0.90 |
| **IPES0163** | 0.43 | 4.00 | 4.00 | 0.66 | 0.00 | 0.59 | 1.00 |
| **IPES0198** | 0.31 | 6.00 | 6.00 | 0.77 | 0.00 | 0.73 | 1.00 |
| **IPES0052** | 0.41 | 5.00 | 4.00 | 0.67 | 0.01 | 0.61 | 0.98 |
| **PGIRD57** | 0.33 | 7.00 | 5.00 | 0.76 | 0.05 | 0.72 | 0.93 |
| **IPES0068** | 0.21 | 9.00 | 7.00 | 0.84 | 0.02 | 0.82 | 0.98 |
| **IPES0098** | 0.32 | 5.00 | 5.00 | 0.75 | 0.00 | 0.70 | 1.00 |
| **PSMP2001** | 0.28 | 13.00 | 8.00 | 0.78 | 0.17 | 0.75 | 0.79 |
| **IPES0066** | 0.41 | 4.00 | 4.00 | 0.71 | 0.00 | 0.67 | 1.00 |
| **PSMP2063** | 0.30 | 6.00 | 6.00 | 0.80 | 0.00 | 0.78 | 1.00 |
| **IPES0102** | 0.63 | 3.00 | 3.00 | 0.48 | 0.00 | 0.39 | 1.00 |
| **PSMP2050** | 0.55 | 4.00 | 4.00 | 0.59 | 0.01 | 0.52 | 0.98 |
| **PSMP2211** | 0.71 | 3.00 | 3.00 | 0.44 | 0.00 | 0.38 | 1.00 |
| **Mean** | **0.45** | **7.33** | **5.28** | **0.66** | **0.06** | **0.62** | **0.91** |

**Supplementary Table 7: Pooled ANOVA for Rainy season 2024 and Summer season 2025**

| **Source of Variation** | **df** | **Mean Sum of Square (MSS)** | | | | | | | | | | | | | | | | |
| --- | --- | --- | --- | --- | --- | --- | --- | --- | --- | --- | --- | --- | --- | --- | --- | --- | --- | --- |
|  |  | **Plant height** | **Leaf length** | **Leaf width** | **Flag leaf area** | **Leaf area** | **Spike length** | **Spike Girth** | **DTFF** | **DTM** | **Productive tillers/pt.** | **Stem Girth** | **L:S Ratio** | **Nodes/pt.** | **GFY1** | **GFY 2** | **GFY3** | **DFY 1** |
| **Block/Replication*Season** | 18 | 644.42 | 50.30 | 0.11 | 49.65 | 2.97 | 3.29 | 2.97 | 5.656* | 1.618* | 0.617 | 0.415 | 0.005 | 0.556 | 0.244 | 0.025 | 0.018 | 0.008 |
| **Replication/Season** | 2 | 1396.77* | 1005.62* | 0.12 | 57.32 | 58.46 | 5.77 | 58.46** | 74.05* | 5.112* | 2.643* | 0.448 | 0.005 | 4.364* | 1.484* | 0.213* | 0.036 | 0.023* |
| **Season** | 1 | 57624.24* | 3770.083* | 23.57** | 1860.45** | 96.57 | 126.72** | 96.57** | 3.24 | 7665.002* | 3.525* | 105.298** | 10.352** | 409.84** | 59.529* | 21.128** | 0.23** | 1.432** |
| **Genotype** | 99 | 2003.21** | 189.39** | 0.84** | 5616.85** | 69.02** | 72.12** | 69.02** | 42.218* | 5.263* | 18.601** | 9.642** | 0.067** | 4.801** | 21.2** | 7.527** | 2.175** | 1.251** |
| **Genotype*Season** | 99 | 1511.83* | 106.46* | 1.11** | 340.76** | 4.93** | 8.85 | 4.93 | 4.048* | 1.321* | 1.552** | 8.662** | 0.054** | 3.787** | 0.386** | 0.131** | 0.081** | 0.036** |
| **Error** | 180 | 429.85 | 42.52 | 0.07 | 60.38 | 7.40 | 8.65 | 7.40 | 1.951 | 0.762 | 0.804 | 0.8 | 0.003 | 0.806 | 0.22 | 0.066 | 0.024 | 0.005 |

| **Source of Variation** | **df** | **Mean Sum of Square (MSS)** | | | | | | | | | | | | | | | |  |
| --- | --- | --- | --- | --- | --- | --- | --- | --- | --- | --- | --- | --- | --- | --- | --- | --- | --- | --- |
|  |  | **DFY 2** | **DFY 3** | **Chlorophyll Content** | **Regeneration 1%** | **Regeneration 2%** | **Canopy Temp.** | **Total Soluble Solids** | **Root Proj. area** | **Total root length** | **Roots tips** | **Forks** | **Segments** | **Max. root diameter** | **Average root diameter** | **Est. root vol.** | **Primary root length** | |
| **Block/Replic**  **ation*Season** | 18 | 0.002 | 0.001 | 12.237 | 57.834 | 81.061 | 9.486 | 0.094 | 6.679 | 243.524 | 1164.574 | 600.191 | 5700.339 | 0.017 | 0.001** | 1.436 | 0.695 | |
| **Replication/**  **Season** | 2 | 0.013* | 0.004* | 1.718 | 714.108* | 449.335* | 16.896 | 0.005 | 8.879 | 2948.274** | 20066.747** | 29990.563** | 200258.411** | 0.168** | 0.017** | 40.808* | 0.174 | |
| **Season** | 1 | 0.726** | 0.421** | 41.738 | 44.669 | 15022.179** | 98.079** | 0.601** | 36.47 | 3409.567** | 3805.718 | 33196.293** | 189413.402** | 0.055 | 0.02** | 127.78** | 0.332 | |
| **Genotype** | 99 | 0.239** | 0.095** | 257.735*** | 4177.846** | 691.097** | 9.698* | 4.644** | 808.025** | 23787.567** | 68403.653** | 55009.964** | 313132.629** | 1.112** | 0.044** | 2042.697** | 102.416** | |
| **Genotype***  **Season** | 99 | 0.006** | 0.002** | 19.795 | 155.27 | 173.401** | 7.467 | 0.109* | 10.194 | 243.674 | 761.413 | 527.94 | 4639.499 | 0.022 | 0.01* | 16.408** | 2.376 | |
| **Error** | 180 | 0.002 | 0.001 | 16.164 | 129.106 | 84.753 | 6.955 | 0.07 | 9.655 | 316.537 | 1046.751 | 509.203 | 4960.129 | 0.027 | 0 | 2.927 | 2.152 | |
